# Meiotic susceptibility for induction of sperm with chromosomal aberrations in patients receiving combination chemotherapy for Hodgkin lymphoma

**DOI:** 10.1371/journal.pone.0242218

**Published:** 2020-12-28

**Authors:** Sara Frias, Paul Van Hummelen, Marvin L. Meistrich, Andrew J. Wyrobek

**Affiliations:** 1 Health Effects Genetics Division, Lawrence Livermore National Laboratory, Livermore, California, United States of America; 2 Laboratorio de Citogenética, Instituto Nacional de Pediatría /Instituto de Investigaciones Biomédicas, Universidad Nacional Autónoma de México, Mexico City, Mexico; 3 Department of Medicine, Stanford University, Palo Alto, California, United States of America; 4 Department of Experimental Radiation Oncology, University of Texas M. D. Anderson Cancer Center, Houston, Texas, United States of America; 5 Biological Systems and Engineering Division, Lawrence Berkeley National Laboratory, Berkeley, California, United States of America; Universite Clermont Auvergne, FRANCE

## Abstract

Improvements in survival rates with gonad-sparing protocols for childhood and adolescence cancer have increased the optimism of survivors to become parents after treatment. Findings in rodents indicate that chromosomal aberrations can be induced in male germ cells by genotoxic exposures and transmitted to offspring and future generations with effects on development, fertility and health. Thus, there is a need for effective technologies to identify human sperm carrying chromosomal aberrations to assess the germ-line risks, especially for cancer survivors who have received genotoxic therapies. The time-dependent changes in the burden of sperm carrying structural chromosomal aberrations were assessed for the first time in a cancer setting, using the AM8 sperm FISH protocol which simultaneously detects abnormalities in chromosomal structure and number in sperm. Nine Hodgkin lymphoma (HL) patients provided 20 semen samples before, during, and after NOVP therapy (Novantrone, Oncovin, Velban and Prednisone) and radiation therapy that produced scattered gonadal doses from <0.05 to 0.6 Gy. Late meiosis was found to be the most sensitive to NOVP treatment for the production of sperm with chromosomal abnormalities, both in structure and number. Earlier stages of spermatogenesis were less sensitive and there was no evidence that therapy-exposed stem cells resulted in increased frequencies of sperm with abnormalities in chromosomal structure or number. This indicates that NOVP therapy may increase the risks for paternal transmission of chromosomal structural aberrations for sperm produced 32 to 45 days after a treatment with these drugs and implies that there are no excess risks for pregnancies conceived more than 6 months after this therapy. This clinical evaluation of the AM8 sperm FISH protocol indicates that it is a promising tool for assessing an individual’s burden of sperm carrying chromosomal structural aberrations as well as aneuploidies after cancer therapy, with broad applications in other clinical and environmental situations that may pose aneugenic or clastogenic risks to human spermatogenesis.

## Introduction

Survival rates for cancers in children and young adults have improved substantially with growing opportunities to regain fertility and consider becoming a parent [1]. However, anti-cancer therapies typically include radiation and drugs that are known or suspected to be aneugenic, clastogenic and mutagenic in rodent model systems. This raises concerns for abnormal pregnancies, birth defects, as well as heritable mutations and translocations in exposed individuals [2]. The agents tested for germ cell mutagenicity in male rodents indicate that chromosomal damage to germ cells is a major heritable concern and that the stages of spermatogenesis that are susceptible to the induction of chromosomal damage vary across drugs [3, 4]. In males, mitotic and meiotic divisions are primary locations for the induction of aneuploidy, while the full duration of spermatogenesis, from stem cell to seminal sperm, is a potential target for the induction of chromosomal structural aberrations and mutations.

Fertilization with sperm carrying chromosomal aberrations poses significant developmental and transgenerational risks. Studies in mice have demonstrated that (1) fertilization with sperm carrying chromosomal aberrations can result in zygotes carrying that chromosomal defect, and (2) subtypes of chromosomal aberrations transmitted by the fertilizing sperm to the zygote are predictive for increased risks for early embryo lethality or increased risks for survival to birth with an abnormal chromosome complement [5]. Most aneuploid embryos die early in development but there are a few exceptions. In human, trisomies of chromosomes 13, 18 and 21 as well as sex chromosome aneuploidies are likely to result in live births, but with phenotypic defects that impact the health and survival of the offspring. Among human pregnancies and live births, autosomal aneuploidies are strongly biased towards maternal origins while some sex-chromosomal aneuploidies have substantial paternal contributions. [6]. Pregnancies and live-births carrying *de novo* balanced translocations are biased towards paternal origin [7, 8], while de novo microdeletions/microduplications are biased towards maternal origins [9, 10]. Pregnancies with unstable or unbalanced aberrations typically die early in pregnancy or suffer malformations. In contrast, pregnancies carrying balanced reciprocal translocation typically result in normal healthy births, but male offspring are predisposed to infertility. Importantly, carriers of chromosomal rearrangements are at increased risks to transmit their chromosomal aberration, in balanced and unbalanced configurations, to future generations.

Surveys of pregnancy outcomes after cancer therapies have provided no conclusive evidence for detrimental effects [11], but the strength of these reports are severely limited by low statistical powers, the unrelated grouping of cancers and therapies, and inclusion of pregnancies resulting from broad ranges of post-therapy times to conception. All human studies with significant numbers of pregnancies and births involved relatively long intervals between the end of therapy and conception so that the sperm that produced the fertilized egg were all derived from germ cells that were stem spermatogonia at the time of therapy. To obtain sufficient sample sizes at short intervals after therapy, methods for the direct analyses of genomic damage within nuclei of individual sperm are needed. The human-sperm/hamster-egg method [12, 13] first allowed inspection of full haploid sperm karyotypes and provided the first evidence that cancer therapy could increase the proportions of sperm with aneuploidies and chromosomal structural aberrations [14, 15]. However, this method was labor-intensive, studies involved only between 25 and 200 sperm per sample, and was limited in the ability to quantify chromosomal duplications, deletions, or translocations.

Fluorescence *in situ* hybridization (FISH) of ejaculated human spermatozoa was a break-through technology for detecting sperm carrying aneuploidies for specific chromosomes in humans and rodent models [16–20] providing new insights into pregnancy risks in medical and experimental settings [10, 11, 14–36]. FISH protocols have confirmed that during and immediately after cancer therapies, aneuploidy was induced in the patients’ sperm, but this returned to baseline levels within 1 to 2 years after therapy [21, 26–30].

Multi-color FISH technology was then further adapted for new protocols to detect human sperm carrying various types of chromosomal structural aberrations. The AM16 sperm FISH protocol [31], ACM protocol [32], and the AM8 protocol [33] all involve multiple probes along chromosome 1 to detect chromosomal rearrangements, breaks, structural duplications and deletions. The AM8 and AM16 protocols also included a probe for another autosome to detect aneuploid sperm within the same assay. These human sperm FISH assays for structural aberration assays were validated by showing that they gave comparable values for structural aberrations as the human-sperm/hamster-egg method with semen from normal men [13, 34, 35] and with semen from translocation carriers [36]. These human sperm FISH protocols for structural chromosomal aberrations have been applied to investigate the chromosomal risks in men with oligospermia [37], advancing age [38] and occupational exposure to benzene [39]. However, none of these assays have been applied to cancer patients.

This study is the first to apply a sperm FISH assay to characterize the time-course of induction and persistence of sperm carrying chromosomal structural aberrations for individuals with a well characterized exposure that is limited in duration, allowing repeated semen analyses to differentiate between susceptible and resistant stages of spermatogenesis. For exposure regimens that induce an increase in chromosomally defective sperm during therapy, it is also important to examine their persistence after the end of treatment, because genomic damage to stem cells, should it occur, can result in permanent production of genetically defective sperm. Here, we report the results from Hodgkin lymphoma (HL) patients, who provided semen samples before treatment, and/or during treatment, and/or at various times after treatment with combination NOVP chemotherapy and radiotherapy, up to 3.5 years for one patient. The NOVP regimen consists of the topoisomerase inhibitor, Novantrone (mitoxantrone); two microtubule polymerization inhibitors, Oncovin (vincristine) and Velban (vinblastine), and Prednisone. NOVP was usually followed by abdominal radiation therapy, which produced between 0.15 and 0.64 Gy scattered radiation to the testes of our patient cohort. Using the AM8 sperm FISH protocol, we characterized the most sensitive window of induction and time course of recovery of sperm carrying numerical and structural chromosomal aberrations in treated patients.

## Results

**Fig 1:**
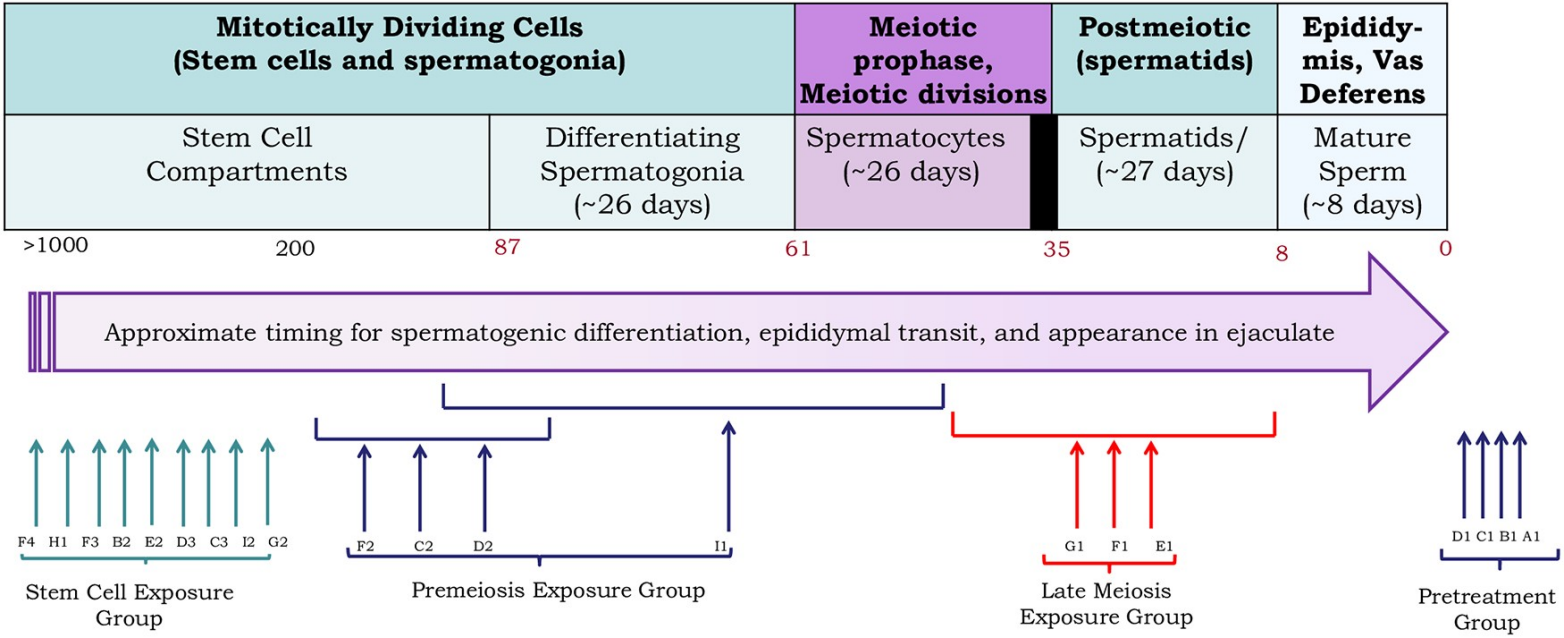
Timing of semen collection showing the stages of spermatogenesis at which the sperm in the ejaculate were during the patients’ treatment with NOVP. The days noted for exposure of cells as spermatogonia, spermatocytes, spermatids, and mature sperm are based on the timing of cells exposed to an administered agent at these stages to appear as sperm in the ejaculate (see S1 Fig in [42]). This timing is based on the kinetics of spermatogenesis in the testis [59], an updated system for staging cells in human spermatogenesis [60], and the appearance of labeled sperm in the ejaculate [61]. Although the state of knowledge of the kinetics of the human spermatogonial compartments is still in a state of flux [62], we selected the time for sperm exposed as stem cells to appear in the ejaculate (87 days) as the time after chemotherapy when sperm count returns to pretreatment levels after chemotherapies with agents that kill differentiating spermatogonia but not stem cells [40,63]. The individual vertical arrows and brackets are aligned with cell types exposed to NOVP treatment for each sample or groups of patients; samples A1, B1, C1 and D1 were collected before the patients started treatment. Table 1 provides patient-specific information on the start and end of NOVP therapy as well doses and durations of gonadal exposure to ionizing radiation. The black box at the end of the in spermatocytes phase (at ~35 days) represents the two meiotic divisions that occur as the male germ cells differentiate into haploid spermatids and subsequently progress through spermiogenesis.

**Fig 2:**
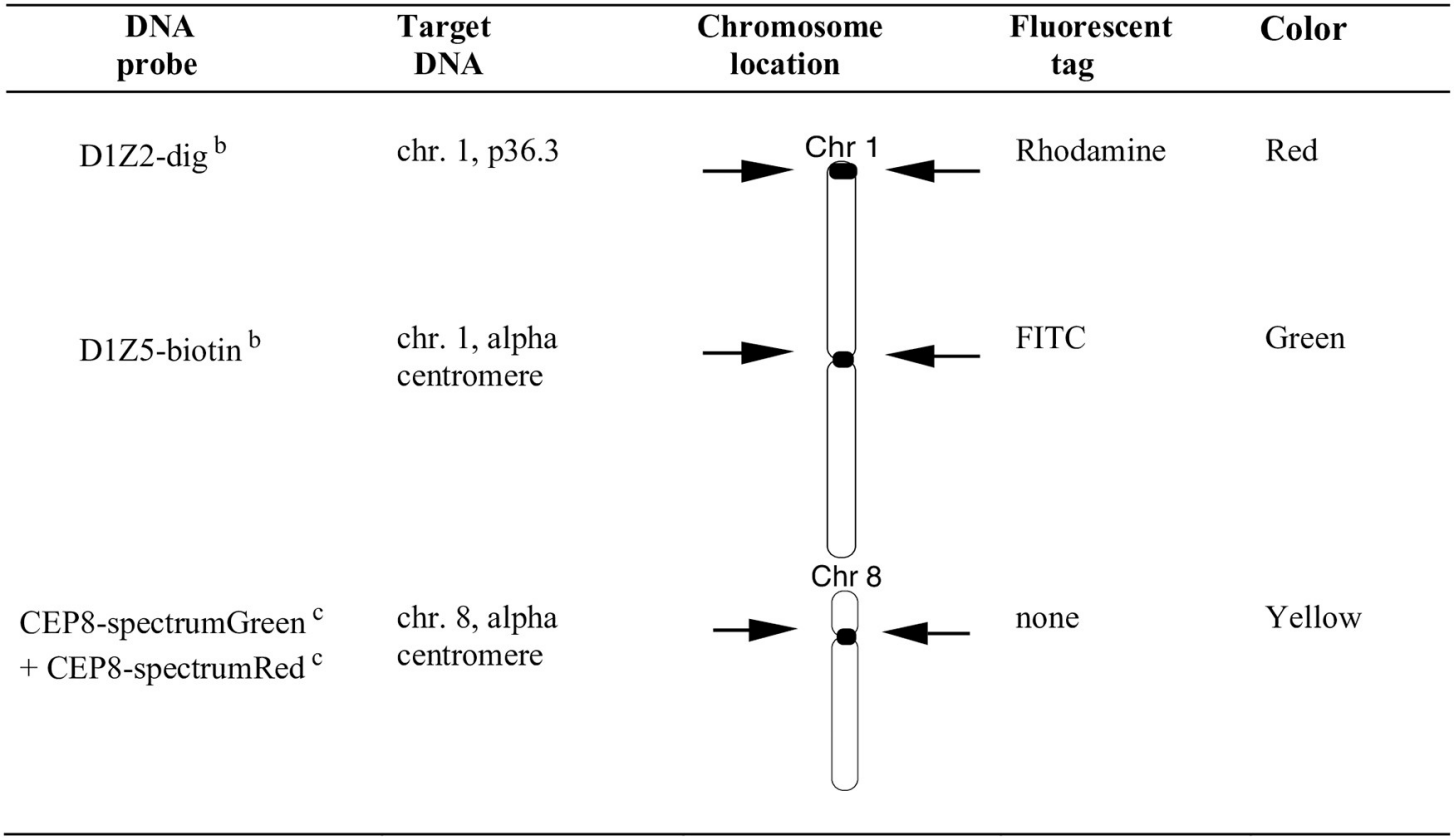
DNA probe and multi-color labeling strategy for the human AM8 sperm FISH assay. This fluorescence labeling strategy was used for the simultaneous detection of sperm carrying duplications or deficiencies in chromosome arm 1p, aneuploidies involving chromosomes 1 and 8, sperm diploidy and complex chromosomal rearrangements (see Methods for scoring details). Approximately 10,000 sperm nuclei were scored per semen sample and nuclei were scored only when a sperm tail could be detected under phase contrast illumination. ^a^ (Van Hummelen *et al*., 1996); ^b^ Oncor (Gaithersburg, MD), ^c^ Vysis (Downer Grove, IL).

Table 1 and Fig 1 describes the HL patient cohort, the treatment details for each patient, the times of semen collection in relation to treatment, and the individual semen cytology results. Both NOVP chemotherapy [40] and abdominal radiation [41] are known to produce major but reversible reductions in sperm count. In our cohort, the comparison of the pre- and post-treatment (stem-cell exposed) groups of samples (Table 1) shows that NOVP plus radiation therapy did not cause a persistent reduction in sperm concentration nor motility. All semen specimens listed on Table 1 were evaluated by the AM8 sperm FISH protocol, as illustrated in Fig 2.

**Table 1:** Twenty semen samples from nine Hodgkin Lymphoma patients, their individual therapy plans, timing of semen specimens, and semen parameters.

Patient Code^a^	Stage	Age^b^	Radiation Therapy ^c^		Sample Code	Semen collection in Days after				Semen parameters		Exposure Groups
			Abdominal field ^d^	Gonadal dose (Gy)		Chemotherapy		Abdominal RT		Sperm Concentration (10^6^/ml)	Sperm Motility (%)	
						Start ^d^	End	Start ^d^	End			
A	IIEB	40	none	0	A-1	pre		none		45.0	71	Pre-treatment
B	IA	40	pre	0	B-1	pre		pre		204.0	74	Pre-treatment
C	IIB	30	pre	0	C-1	pre		pre		28.8	76	Pre-treatment
D	IA	27	none	0	D-1	pre		none		50.2	69	Pre-treatment
E	IIA	31	pre	0	E-1	41	-6	pre		16.8	69	Late Meiosis
F	IIA	27	pre	0	F-1	45	-7	pre		13.7	64	Late Meiosis
G	IIA	36	pre	0	G-1	49	-7	pre		61.6	37	Late Meiosis
C	IIB	30	U-2/3	0.26	C-2		110	28	0	14.5	55	Premeiosis
D	IA	27	none	<0.05	D-2	134	85			11.4	25	Premeiosis
F	IIA	27	U-2/3	0.15–0.39	F-2		154	58	30	12.3	70	Premeiosis
I	III2A	33	Spade	0.64	I-1	107	45	41	0	27	64	Premeiosis
B	IA	40	U-2/3	0.15–0.39	B-2		585		470	83.5	65	Stem cells
C	IIB	30	U-2/3	0.26	C-3		494		384	38.4	90	Stem cells
D	IA	27	none	<0.05	D-3		546			4.0	38	Stem cells
E	IIA	31	U-2/3	0.15–0.39	E-2		549		379	64.2	43	Stem cells
F	IIA	27	U-2/3	0.15–0.39	F-3		614		490	34.2	76	Stem cells
F	IIA	27	U-2/3	0.15–0.39	F-4		1233		1109	213.0	66	Stem cells
G	IIA	36	U-2/3	0.15–0.39	G-2		231		107	9.7	48	Stem cells
H	IIB	28	U-2/3	0.21	H-1		936		817	40.4	80	Stem cells
I	III2A	33	Spade	0.64	I-2		464		419	6.8	76	Stem cells

Samples are grouped by the stages of spermatogenesis at which the spermatozoa were at the time of treatment.

^a^Patients E, F, and G provided repeated specimens (see Fig 2)

^b^ Age at first treatment.

^c^ Specimens with a range of doses are estimates based on TLD measurements in similarly treated patients.

^d^ “pre” refers to specimens provided before the start of indicated therapy. Start of treatment is not given when the end is >100 days since the sperm are all derived from cells treated as stem cells.

### Sperm with structural chromosomal abnormalities

**Fig 3:**
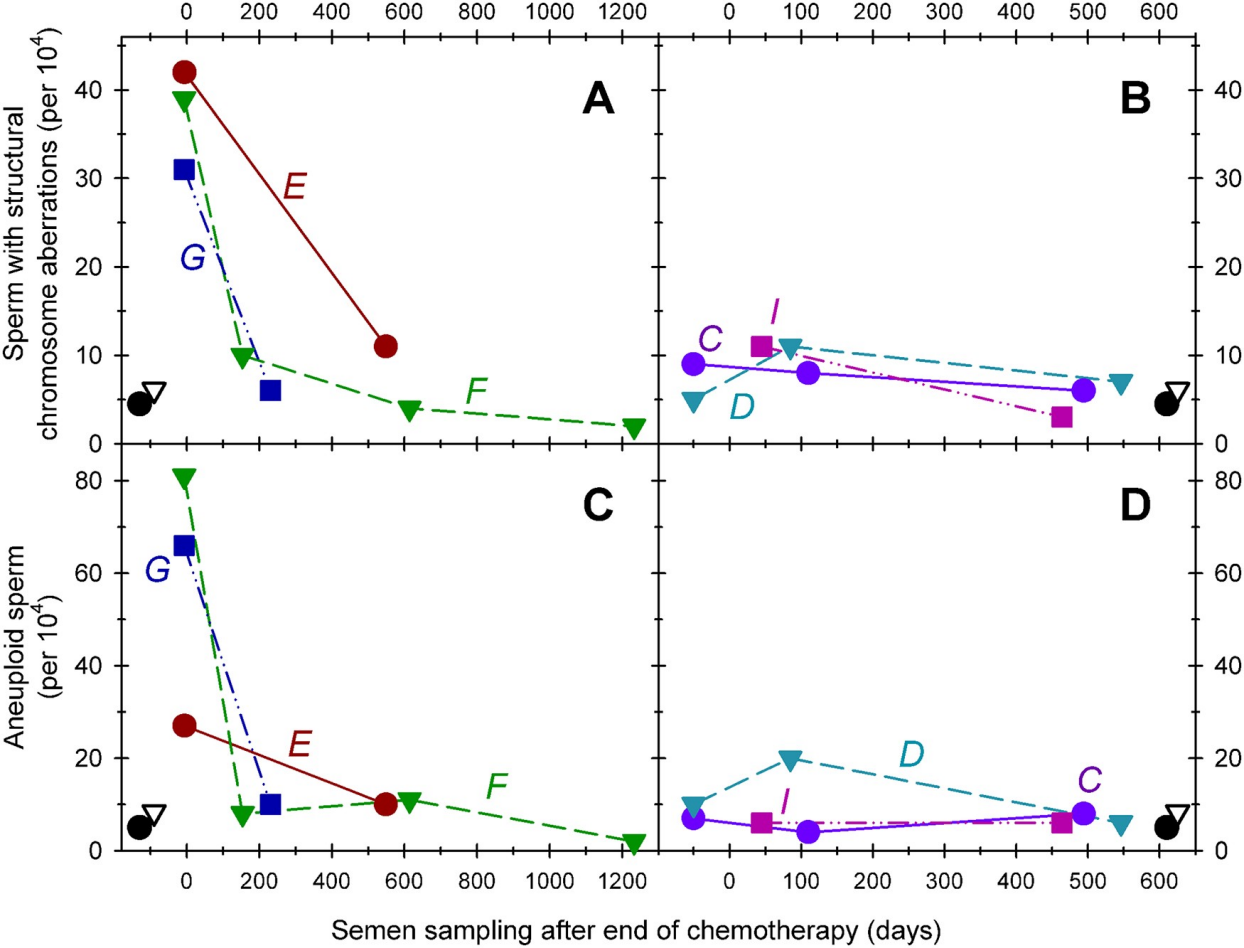
Effects of time after NOVP treatment on the fractions of sperm carrying structural aberrations (Panels A, B) or numerical abnormalities (Panel C, D). Six HL patients (C, D, E, F, G, I Table 1) provided repeated semen specimen at various times before, during, and after treatment with NOVP. Zero time represents the end of chemotherapy; the samples plotted at negative times in panels B and D represents that patient’s pretreatment value. The Y-axis represents the frequency of cells with chromosomal abnormalities (per 10,000 cells examined). Straight lines connect values of sperm samples donated by the same patient at various times during and after treatment and the patients are identified by the italicized letters next to the plot. The black symbols in the lower left or right corners represent the average values for historical controls (open triangles) and the pretreatment group (filled circles).

**Fig 4:**
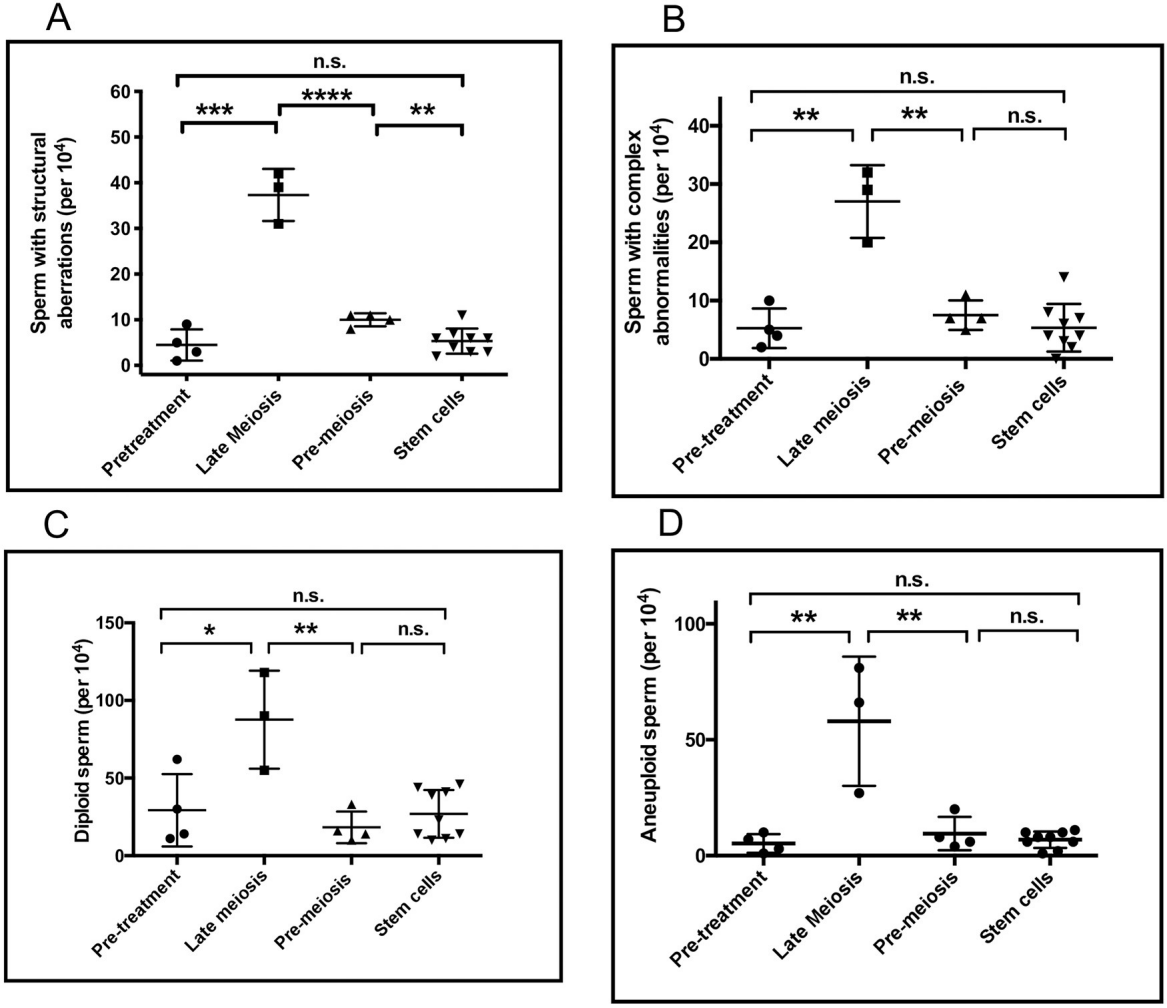
Effects of time after NOVP treatment on the fraction of sperm carrying specific classes of chromosomal abnormalities. Structural chromosomal aberrations (Panel A), sperm with complex chromosomal abnormalities (Panel B), diploid sperm (Panel C), aneuploid sperm (Panel D). Semen specimens were assigned to an exposure group (Fig 1 and Table 1). Statistical analyses are based on one-way ANOVA and post hoc tests with p values: * <0.05, **<0.01, ***<0.005, **** 0.001, no marking = Not significant.

Pretreatment fractions of sperm with chromosomal structural abnormalities were in range of earlier published data of normal healthy controls [33]. The effects of NOVP therapy on the frequencies of sperm with chromosomal structural aberrations are summarized in Table 2. ANOVA of the four analyses groups indicated a strong effect of NOVP treatment (F(3, 16) = 81.8, p<0.0001). As shown in Fig 4a, the late meiosis exposure group had significantly higher frequencies of sperm carrying partial duplications and deletions involving chromosome 1p (unpaired t test, p = 0.0002), compared to the pretreatment group. The effect of treatment remained slightly elevated in the pre-meiosis exposure group compared to both the pretreatment and stem cell groups (p = 0.002). The transient nature of the NOVP-induced frequencies of sperm with chromosomal structural aberrations was confirmed by AM8 analyses of repeated specimens provided over time by patients E, F and G (Table 2, Fig 3A). The fraction of sperm with structural aberrations in the stem cell exposure group was not different from the baseline values in the pretreatment group.

**Table 2:** AM8 FISH analyses of sperm carrying structural chromosomal aberrations in Hodgkin Lymphoma patients before, during and after treatment with NOVP chemotherapy^a^.

Exposure Groups Specimen Codes	duplication 1p	deletion 1p	Sum	% extrapolated genome-wide
Historic controls^b^	3.2 (1.9)	2.9 (3.6)	6.1 (4.2)	1.5%
Pre-treatment				
A-1	2	1	3	
B-1	0	1	1	
C-1	7	2	9	
D-1	5	0	5	
mean	3.5 (3.1)	1.0 (0.8)	4.5 (3.4)	1.1%
Late Meiosis Exposure				
E-1	29	13	42	
F-1	13	26	39	
G-1	27	4	31	
Mean	23.0 (8.7)	14.3 (11.1)	37.3 (5.7) ^c^	9.3%
Premeiosis Exposure				
C-2	8	0	8	
D-2	5	6	11	
F-2	5	5	10	
I-1	9	2	11	
Mean	6.8 (2.8)	3.3 (2.4)	10.0 (3.6) ^d^	2.5%
Stem Cell Exposure				
B-2	3	0	3	
C-3	5	1	6	
D-3	7	0	7	
E-2	8	3	11	
F-3	2	2	4	
F-4	2	0	2	
G-2	4	2	6	
H-1	5	1	6	
I-2	1	2	3	
Mean	4.1 (2.3)	1.2 (1.0)	5.3 (2.6)	1.3%

^a^ Frequency of abnormal sperm per 10 000 sperm (SD)

^b^ 3 healthy donors, 120,686 sperm (Van Hummelen *et al*., 1996)

p values compared to pretreatment group:

^c^<0.005,

^d^ <0.01, no marking = Not significant

### Sperm with complex chromosomal abnormalities

NOVP treatment also induced transient elevations in the frequencies of sperm with complex chromosomal abnormalities, as described in methods (Table 3, Fig 4b). ANOVA indicated significant effects of treatment (F(3,16) = 23.2, p<0.0001). The late meiosis exposure group had significantly higher frequencies of sperm with complex abnormalities (p = 0.002), compared to the pre-treatment group. The frequencies of sperm with complex abnormalities in the pre-meiosis and stem cell exposure groups were not significantly different from pretreatment baselines, consistent with full recovery to baseline frequencies.

**Table 3:** AM8 FISH analyses of sperm carrying complex chromosomal abnormalities or sperm diploidy in Hodgkin lymphoma patients before, during and after treatment with NOVP chemotherapy^a^.

Exposure Groups Specimen Codes	Complex abnormalities	Sperm diploidy
Historic controls^b^	2.9 (2.6)	6.6 (4.0)
Pretreatment		
A-1	4	11
B-1	2	14
C-1	10	62
D-1	5	30
Mean	5.5 (3.4)	29.2 (23.3)
Late Meiosis exposure		
E-1	20	118
F-1	29	55
G-1	32	90
Mean	27.0 (6.2) ^c^	87.7 (31.6) ^c^
Premeiosis exposure		
C-2	7	33
D-2	11	16
F-2	7	14
I-1	5	10
Mean	7.5 (2.5)	18.2 (10.1)
Stem-Cell exposure		
B-2	6	23
C-3	8	41
D-3	7	39
E-2	14	44
F-3	4	14
F-4	0	10
G-2	4	46
H-1	2	11
I-2	3	14
Mean	5.3 (4.1)	28.5 (15.5)

^a^ Frequency of abnormal sperm per 10 000 sperm (SD)

^b^ 3 healthy donors, 120,686 sperm (Van Hummelen *et al*., 1996).

p values compared to pretreatment group:

^c^<0.005, no marking = Not significant P>0.05

### Sperm with abnormalities in chromosome numbers

#### Diploid sperm

NOVP treatment also induced transient elevations in the frequencies of diploid sperm (Table 4, Fig 4c). ANOVA for diploid sperm indicated significant effects of treatment (F(3,16) = 9.3, p<0.0009), without significant variation in standard deviations across the analyses groups. The late meiosis group had significantly higher frequencies of diploid sperm (p = 0.04), compared to the pre-treatment group. The elevations in sperm diploidy declined after treatment, with no significant differences in sperm diploidy between the pre-meiosis, stem cell, and pre-treatment exposure groups. However, donor C showed atypically high frequencies of diploid sperm in his pretreatment sample, which may be associated with systemic symptoms, such as fever, associated with his diagnosis of stage IIB HL disease.

**Table 4:** AM8 FISH analyses of sperm aneuploidy in Hodgkin Lymphoma patients before, during and after treatment with NOVP chemotherapy^a^.

Exposure Groups Specimen Codes	disomy 1	nullisomy 1	disomy 8	nullisomy 8	sum	% extrapolated genome-wide
Historic controls^b^	1.7 (2.2)	0.4 (0.5)	1.9 (2.3)	4.4 (4.8)	8.3	0.9%
Pre-treatment						
A-1	2	1	0	0	3	
B-1	1	0	0	0	1	
C-1	0	0	7	0	7	
D-1	3	4	1	2	10	
Mean	1.5 (1.3)	1.2 (1.9)	2.0 (3.4)	0.5 (1.0)	5.0 (4.0)	0.6%
Late Meiosis exposure						
E-1	9	2	11	5	27	
F-1	17	45	7	12	81	
G-1	22	20	15	9	66	
Mean	16.0 (6.6)	22.3 (21.6)	11.0 (4.0)	8.7 (3.5)	58.0 (27.9)^c^	6.7%
Premeiosis exposure						
C-2	2	0	2	0	4	
D-2	12	5	3	0	20	
F-2	3	2	3	0	8	
I-1	1	1	3	1	6	1.1%
Mean	4.5 (5.0)	2 (2.2)	2.7 (0.5)	0.2 (0.5)	9.5 (7.1)	
Stem cell exposure						
B-2	0	0	1	0	1	
C-3	7	0	1	0	8	
D-3	3	1	1	1	6	
E-2	4	1	4	1	10	
F-3	3	4	2	2	11	
F-4	0	2	0	0	2	
G-2	2	6	2	0	10	
H-1	2	2	3	1	8	
I-2	2	3	1	0	6	
Mean	2.6 (2.1)	2.1 (1.9)	1.7 (1.2)	0.6 (0.7)	6.9 (3.5)	0.8%

^a^ Frequency of aneuploid sperm per 10 000 sperm (SD)

^b^ 3 healthy donors, 120,686 sperm (Van Hummelen *et al*., 1996)

p values compared to pretreatment group:

^c^ <0.005, no marking = Not significant

#### Aneuploid sperm

A side benefit of the AM8 sperm FISH protocol provided a separate analysis of the frequencies of aneuploidies involving chromosomes 1 and 8, and confirmed that NOVP therapy also induced a transient increase in the frequencies of aneuploid sperm (Fig 4d). Table 4 lists the frequencies of aneuploid sperm involving chromosomes 1 and 8 for all patients in our cohort, using the same smears analyzed for sperm with structural aberrations and complex chromosomal abnormalities. Pretreatment levels of sperm aneuploidy were in range of earlier published data of normal healthy men [33] with slight differences in baseline frequencies in two prior studies [21, 27], ANOVA of the four exposure groups indicated significant effects of NOVP treatment on sperm aneuploidy (F(4,28) = 23.1, p<0.0001). The late meiosis exposure group had the highest frequencies of aneuploid sperm (p = 0.01), compared to the pre-treatment group. The elevations in aneuploidy sharply declined after treatment, consistent with full recovery to baseline. Both the pre-meiosis and stem cell exposure groups were not significantly different from the pretreatment values. The transient nature of the NOVP-induced frequencies of aneuploid sperm is confirmed in patients E, F and G who provided repeated specimens over time (Table 4, Fig 3C).

## Discussion

Hodgkin lymphoma patients who were treated with NOVP chemotherapy including scattered gonadal doses from radiotherapy produced transient increases in the fraction of various categories of chromosomally abnormal sperm, as assessed by the AM8 sperm FISH protocol (Fig 2). This protocol simultaneously detects sperm with chromosomal structure aberrations, in addition to aneuploidy, diploidy, and complex chromosomal abnormalities (Tables 2–4).

### Susceptibility of late meiosis and the kinetics to recovery

The differentiation stages of spermatogenesis from stem cells to mature sperm vary substantially in their susceptibilities to transmittable genomic damage from cancer drugs, depending on the mechanisms of action of the exposure agents, dosing regimen, type of genomic damage and other factors [3, 42]. We analyzed semen samples collected at different times relative to the patients’ anticancer treatment, to identify the windows of spermatogenesis that were most susceptible for the induction of chromosomal rearrangements versus aneuploidies and to compare kinetics of recovery for both categories of genomic damage after the end of therapy. The peak increases in the proportions of sperm carrying structural or numerical chromosomal abnormalities occurred in semen collected towards the end of NOVP treatment, before any radiation was given, and corresponded to sperm derived from the germ cells treated with NOVP during late meiosis (Table 1, Fig 1). The data collected by the AM8 protocol can be used to estimate the genome-wide frequencies of sperm carrying abnormalities in chromosome structure or number after NOVP treatment during late meiosis allowing the following assumptions: that damage is randomly distributed across the genome, that the size of chromosome 1p represents ~4% of the genomic target for structural aberrations, and that chromosomes 1 and 8 represents ~9% of the genomic target for numerical abnormalities. Thus, we calculate that after exposure in late meiosis, ~9% of sperm should have structural aberrations (Table 2) plus ~7% of sperm should have numerical abnormalities (Table 4) for a total of about 16% chromosomally abnormal sperm. This represents about a 10-fold increase above the average pretreatment baseline of 1.6%. The estimate of the fraction of sperm with chromosomal abnormalities is likely to be an underestimate because it does not include complex rearrangements or diploidies. Also, treatment-induced damage to post-meiotic spermatids is not detected by the AM8 protocol because this assay requires at least one cell division between drug treatment and sperm analyses. Our findings reinforce the concern that there is increased genetic risk to offspring conceived from sperm obtained during and shortly after chemotherapy.

Semen samples from the pre-meiosis exposure group (Table 1) were provided at later times after NOVP than the late-meiosis group, from 45 to 154 days after the completion of NOVP and at 0–30 days after completion of abdominal radiotherapy. The pre-meiosis group showed no increase in the frequencies of aneuploid sperm above pre-treatment baseline, but showed a significant ~2-fold increase in sperm with structural chromosomal aberrations. Three of the pre-meiosis patients received abdominal radiation shortly after the completion of the NOVP therapy (Table 1). Sample I-1, which had low levels of aneuploid sperm and modest levels of structural abnormalities was informative to further refine the most sensitive germ cell stages. It was provided at 45 days after the end of NOVP treatment (Fig 1), which represents sperm derived from germ cells treated in the window between stem cells and, in the case of mitoxantrone and vinblastine, the zygotene spermatocytes, and for vincristine in early pachytene. The high levels of abnormalities in the late meiosis group and the steep decline in the pre-meiosis group supports the conclusion that the most sensitive window for the induction of sperm carrying structural or numerical chromosomal abnormalities after NOVP therapy is between the middle of the pachytene spermatocyte stage and the completion of the second meiotic division, i.e, in late meiosis.

Although aneuploidies and structural rearrangements arise by different molecular mechanisms, the peak of induction of aneuploidy in late meiosis coincided with the most sensitive window for the induction of abnormalities in chromosome structure. These results are in general agreement with experimental animal data. The microtubule inhibiting agents, vincristine and vinblastine, are potent inducers of aneuploidy, due to aberrant chromosome segregation at meiosis I [43]. Among the drugs in the NOVP cocktail, only mitoxantrone, a topoisomerase II inhibitor, appears to be able to induce DNA strand breaks, which are a pre-requisite for forming structural rearrangements [44]. The ability of a topoisomerase II inhibitor to induce chromosomal abnormalities is supported by induction of aneuploidy and chromosomal structure aberrations in pachytene spermatocytes by treatment of mice with etoposide, and the transmission of these aberrations to the sperm formed and to the zygote at fertilization [45, 46].

The reduction in sperm aneuploidy to baseline levels in samples provided between 45 and 154 days after the end of chemotherapy can be compared with available animal data [45, 46], which showed that preleptotene/leptotene spermatocytes, but not spermatogonia were sensitive to aneuploidy induction by the topoisomerase II inhibitor etoposide. The low level of sperm aneuploidy in I-1 indicates the early (preleptotene/leptotene) human spermatocytes may not be sensitive to aneuploidy induction by mitoxantrone. The low levels of sperm aneuploidy in samples F-2, C-2, and D-2 were consistent with the lack of induction of aneuploidy in murine spermatogonia with etoposide.

In contrast to the aneuploidy data, the average levels of sperm with structural aberrations resulting from pre-meiosis exposure (i.e., the pre-meiosis group) remained significantly above pretreatment values (Table 2, Fig 4A). In mice, etoposide induces levels of structural aberrations in early primary spermatocytes at similar levels as in late meiotic spermatocytes [45] and in type A spermatogonia at low but significant levels [46]. Mitoxantrone may act similarly in the pre-meiosis patient group. Another contributing factor to the slight increase in sperm with structural aberrations in this group may be the exposure of meiotic cells to ionizing radiation in Samples F-2 (meiotic dose ~0.25 Gy) and I-1 (meiotic dose 0.15 Gy), which is known to induce structural chromosomal abnormalities in mouse spermatogonia and spermatocytes [47–49].

### Lack of persistence of chromosomal damage in sperm after NOVP treatment of stem cells

The nine samples assigned to the stem cell group were obtained more than 231 days after NOVP and 107 days after radiation therapy (Figs 1 and 4), and the sperm in these samples were exposed to both NOVP and radiation as stem cells. In our study, the averages for frequencies of numerical and structural abnormalities in the stem cell group were not different from pretreatment and historical control values indicating that neither exposure produced a persistent increase in chromosomally abnormal sperm in our patient cohort. Two previous studies reported ~40% higher overall aneuploidy frequencies in their HL pretreatment samples compared to healthy donors [28, 29]. These studies analyzed different chromosomes (18, 21, X and Y) than our study and reported no significant differences for certain subtypes of aneuploidies. In our study, the lack of significant differences in sperm aneuploidies when comparing the donors, pretreatment HL and stem cell exposure groups may be attributable to lower susceptibilities of chromosomes 1 and 8 in the AM8 assay or may be due to small numbers of participants per group (Tables 3 and 4).

The low levels of chromosomal abnormalities for the stem cell exposure group are consistent with studies in mice. In mice, very few drugs induced transmissible chromosomal rearrangements after treatment of spermatogenic stem cells [2–4]. One exception is etoposide [46] which, by sperm FISH analysis, induced a very low level of chromosomal structural abnormalities in stem cells (only 3.5% of the value induced in spermatocytes). On the other hand, exposure of mouse stem spermatogonia to ionizing radiation can result in transmissible chromosomal aberrations [50, 51], but their incidence was much lower than the frequencies obtained after irradiating spermatocytes or spermatids. The failure to observe an elevated frequency of sperm carrying chromosomal aberrations in patients whose stem cells were exposed to a topoisomerase inhibitor or ionizing radiation may be due to the relatively low doses of gonadal radiation (0 to 0.64 Gy) received by our patients and that any chromosomal aberrations induced in the stem cells of patients may have been filtered out during numerous mitotic and meiotic divisions between treatment and sperm analyses. In contrast to the findings of genomic instability in lymphocytes of HL survivors [52, 53], the frequencies of chromosomal aberrations in sperm from six months to 3 years after therapy, remained in range of pretreatment samples, providing reassuring evidence to patients that these agents do not induce persistent chromosomal damage in sperm after treatment of stem cells [54].

### Clinical relevance and environmental applications

Our finding that NOVP induced transient increases in aneuploid sperm is consistent with previous studies of aneuploidy after other antineoplastic treatment regimens. However the time to return to baseline appears to vary substantially across therapies. After treatment of HL patients with chemotherapy lacking strong alkylating or platinating agents, such as NOVP and ABVD (Adriamycin, bleomycin, vinblastine, dacarbazine), both often combined with abdominal radiation, aneuploidy levels returned to control levels within 6 months or less [21, 29]. Patients with seminoma treated with radiotherapy alone, which in our experience delivers a gonadal dose of about 0.7 Gy [55] showed increases in aneuploidy that lasted for 6 months but decreased to baseline at 12 months [30]. HL patients treated with alkylating agent therapy such as CHOP (cyclophosphamide, Adriamycin, Oncovin, prednisone) or MOPP (mechlorethamine, Oncovin, procarbazine, prednisone) and testicular cancer patients treated with BEP (bleomycin, etoposide, cisplatin) showed increases in the frequencies of sperm aneuploidy that persisted for longer times before reverting to baseline values at 24 months [28–30, 56]

Chromosomal aberrations and aneuploidies occur by different molecular mechanisms and the assessments of both endpoints are necessary to evaluate the relative clastogenic and aneugenic risks to spermatogenic cells. Although in our study the late meiotic cells were most susceptible for the induction of sperm with both structural and numerical aberrations, the premeiotic cells showed an increased level of structural aberrations while numerical abnormalities had returned to baseline in the premeiotic group (Table 2, Fig 4A). Similarly, paternal aging has been associated with increased frequencies of sperm with structural chromosomal aberrations but not aneuploidies [38, 57]. The possibility of differential treatment effects suggests the need to assess both aneuploidies and structural chromosome aberrations for setting guidelines for the period of time that a patient should wait before attempting conception.

Our study is the first clinical application of sperm FISH to simultaneously assess the differential effects of chemotherapy or radiation therapy on the fractions of sperm with aneuploidy and sperm with chromosomal structure aberrations. The AM8 protocol provides new information that the incidence of structural abnormalities in the sperm from the NOVP-treated patients is at least as large as that observed for aneuploidy. Our finding that exposure of spermatogenic stem cells to NOVP or radiation doses of ≤0.64 Gy does not lead to persistent chromosomal damage in sperm will provide some reassurance to survivors who hope to father their own children after therapy.

Our study has limitations. Our findings with NOVP/radiotherapy do not directly extrapolate to other cancer chemotherapies or to higher doses of gonadal radiation, because our study is based on only two genotoxic drugs with mechanisms of action that are not necessarily relevant to other chemotherapies. Also, our time-response findings do not predict the susceptible windows of spermatogenesis nor the time-course of decay of sperm damage after other therapies, noting that chromosomal aberrations have been reported in sperm collected up to 20 years after other therapy [14]. Also, the numbers of patients in our exposure group were small, and few patients who donated repeated samples before, during and after treatment. Our study was not a classic longitudinal study suggesting the need for longitudinal clinical investigations of the time-course of induction and persistence of sperm damage for common cancer therapies.

Our study illustrates the value of combination sperm FISH protocols for the simultaneous detection of chromosomal structural aberrations and aneuploidy in sperm of cancer patients to provide personalized medical information on the magnitude of therapy-induced chromosomal damage in their sperm and time after treatment to return to pre-treatment baseline values. Combination sperm FISH protocols are also promising tools for applications in public health settings to estimate risks to individuals and groups of young men of reproductive age for producing sperm carrying aneuploidy and chromosomal structural defects from common environmental or occupational exposure or health condition of concern.

## Materials and methods

### Patient cohorts and semen collection times in relation to therapies

Twenty semen samples were provided by nine adult patients diagnosed with HL and treated at the University of Texas, M.D. Anderson Center Houston (Table 1). Participants provided written informed consent from the participants to use their samples for the purposes of research. This consent was obtained at the time they were enrolled in the study, which was before the initiation of therapy in some, but not all cases. Seven of the patients were white non-Hispanic, one was Hispanic, and one was of Middle-Eastern ethnicity. None received prior anti-cancer chemotherapy or radiation therapy prior to recruitment into this study. All patients received NOVP-combination chemotherapy consisted of three 21-day cycles of Novantrone (mitoxantrone, 10 mg/m^2^ on day 1); Oncovin (vincristine, 2 mg on day 8); Velban (vinblastine, 6 mg/m^2^ on day 1) and Prednisone (100 mg/day on days 1–5). This research was approved by the Institutional Review Boards of the University of Texas M. D. Anderson Cancer and the Lawrence Livermore National Laboratory.

After completing NOVP therapy, all patients received radiation to a mantle field from which there was less than 0.05 Gy scattered radiation to the gonads. Next, all but one (patient D) received either upper 2/3 abdominal radiation, or the abdominal field supplemented with a downward extending spade field (patient I). The gonadal dose was measured by thermoluminescent dosimetry (TLD) in some patients [58]. A range of gonadal doses for abdominal field therapy (0.15 to 0.39 Gy) was estimated from values for other similarly treated patients who did have TLD measurements [41]. The patient receiving the abdominal spade field (patient I) received a gonadal dose of 0.64 Gy.

Four patients provided semen specimens before any treatment (pre-treatment group, Table 1, Fig 1) and 16 samples were provided at various times after treatments. The 3 patients in the late meiosis exposure group provided specimens from 41 to 49 days from the start of NOVP treatment. This means that the sperm were exposed to mitoxantrone and vinblastine on day 1 of the first course of NOVP between 41 and 49 days before sample collection and exposed to vincristine on day 8, which was between 33 and 41 days before collection. Hence the cells would have been at the middle pachytene to meiotic division stages of spermatogenesis (Fig 1). In addition, the cells were exposed at the spermatid stages during the second course of chemotherapy, but this FISH assay cannot detect chromosomal damage in post-meiotic cells since there is no cell division between that stage and the sperm. All 3 samples were obtained at the start of the third course of NOVP therapy and prior to the radiation treatments. The four patients in the pre-meiosis exposure group provided samples between 45 and 154 days after the end of their NOVP treatment. Sample I-1 was collected at 45 days after the end of NOVP treatment so that the sperm were primarily exposed as differentiating spermatogonia and early spermatocytes. Sample D-2 was collected at 85 days after the end of NOVP treatment so that the sperm had little exposure as differentiating spermatogonia but mostly as stem cells about to initiate differentiation. The sperm in the other two premeiosis samples (C-2 and F-2) were obtained from patients exposed to the chemotherapy when they were in the later stages of the stem cell compartment. Three of the patients in the premeiosis group also received abdominal radiotherapy prior to sample collection. The cells that produced the sampled sperm were exposed to radiation as spermatids and sperm (C-2), spermatocytes (F-2), and as late spermatocytes through sperm (I-1). The 9 specimens of the stem cell analysis group were provided between 231 to 1233 days after the end of NOVP chemotherapy (107 to 1109 days after the end of abdominal radiotherapy) so that the sperm in these ejaculates were stem cells during both the chemo and radiation treatments.

### Semen analyses, AM8 sperm FISH protocol, and scoring criteria

Specimens were delivered to the clinic within 2 hours of collection and assessed by conventional semen analyses (concentration and motility, Table 1) as described previously [40]. Samples were frozen at -20°C without added media and shipped on dry ice to Lawrence Livermore National Laboratory and stored at -80°C until use. Sperm smears were processed using the AM8 protocol with a cocktail of DNA probes for the centromeres of chromosome 1 and 8 and the 1p telomere of chromosome 1 (Fig 2) [33, 36]. The probes of the distal part of the p arm and the centromere of chromosome 1 were labeled with Digoxigenin or Biotin, respectively, and tagged with red or green labeled fluorescent antibodies, respectively. Two probes for chromosome 8, directly labeled with spectrum green or spectrum orange, were combined giving a yellow color. The slide preparation and hybridization protocols were described previously [33]. Approximately 10,000 sperm nuclei were scored per semen sample using the AM8 protocol, with slides coded by a person not involved in scoring. Chromosomally normal sperm carry one signal of each chromosomal target: chromosome 1 centromere (D1Z5, Oncor, Gaithersburg, MD) in green, chromosome 8 centromere (CEP8, Vysis, Downers Grove, IL) in yellow, and chromosome arm 1p (D1Z2, Oncor, Gaithersburg, MD) in red, as illustrated in Fig 2. Sperm that carry a structural aberration involving chromosome arm 1p were identified as having one signal for chromosome 1 centromere (D1Z5), one for chromosome 8 centromere (CEP8) and none or two signals of chromosome arm 1p (D1Z2). Several categories of aneuploid sperm are detected by the AM8 protocol. Aneuploid sperm that are disomic for chromosome 1 carry two signals of D1Z2, two for D1Z5 plus one signal for CEP8. Aneuploid sperm that are nullisomic for chromosome 1 carry no signals of D1Z2 or D1Z5 plus one signal for CEP8. Aneuploid sperm that are disomic for chromosome 8 carry two signals of CEP8, one signal of D1Z2 and one signal for D1Z5. Aneuploid sperm that are nullisomic for chromosome 8 carry no signal of CEP8, plus one signal of D1Z2 and one signal for D1Z5. Sperm carrying a full diploid complement of chromosomes carry two signals of each probe (and the nuclei are generally larger in size). All other permutations of the presence or absence of the probes, which could not be assigned to any of the above phenotypes, were classified as sperm with complex aberrations. Nuclei were scored only when a sperm tail could be detected under phase contrast illumination.

### Data analyses

The effects of therapy on semen of our HL patient cohorts were evaluated using PRISM 6.0 software and other tools applied to four analyses groups (Table 1: pretreatment, late meiosis, pre-meiosis, and stem cell). One-way ANOVA was performed across the four groups followed by Brown-Forsythe or Bartlett’s tests for the significance of variations in standard deviations across the groups. Significant ANOVAs were followed by two-tailed post-hoc testing, using Welch-corrected t tests for ANOVA findings with significant variation in standard deviations among the groups, or with t-test without correction for cases that did not show significant variation in the standard deviations across the groups. The non-parametric Mann-Whitney test was applied for confirmation of marginal findings. Prism graphing tools were applied to show values for individual specimen values by group, and error bars represented means and SDs.
